# Machine learning-enhanced gesture recognition through impedance signal analysis

**DOI:** 10.2478/joeb-2024-0007

**Published:** 2024-06-11

**Authors:** Hoang Nhut Huynh, Quoc Tuan Nguyen Diep, Minh Quan Cao Dinh, Anh Tu Tran, Nguyen Chau Dang, Thien Luan Phan, Trung Nghia Tran, Congo Tak Shing Ching

**Affiliations:** 1 Laboratory of Laser Technology, Ho Chi Minh City University of Technology (HCMUT), Ho Chi Minh City 72409, Vietnam; 2 Laboratory of General Physics, Ho Chi Minh City University of Technology (HCMUT), Ho Chi Minh City 72409, Vietnam; 3 Department of Telecommunication Engineering, Ho Chi Minh City University of Technology (HCMUT), Ho Chi Minh City 72409, Vietnam; 4 Vietnam National University Ho Chi Minh City, Linh Trung Ward, Thu Duc, Ho Chi Minh City 71308, Vietnam; 5 Graduate Institute of Biomedical Engineering, National Chung Hsing University, Taichung 402, Taiwan; 6 International Doctoral Program in Agriculture, National Chung Hsing University, Taichung 402, Taiwan; 7 Department of Electrical Engineering, National Chi Nan University, Puli Township 54561, Taiwan

**Keywords:** Bio-impedance, Gesture Recognition, Impedance Signal Spectrum Analysis (ISSA), Machine Learning

## Abstract

Gesture recognition is a crucial aspect in the advancement of virtual reality, healthcare, and human-computer interaction, and requires innovative methodologies to meet the increasing demands for precision. This paper presents a novel approach that combines Impedance Signal Spectrum Analysis (ISSA) with machine learning to improve gesture recognition precision. A diverse dataset that included participants from various demographic backgrounds (five individuals) who were each executing a range of predefined gestures. The predefined gestures were designed to encompass a broad spectrum of hand movements, including intricate and subtle variations, to challenge the robustness of the proposed methodology. The machine learning model using the K-Nearest Neighbors (KNN), Gradient Boosting Machine (GBM), Naive Bayes (NB), Logistic Regression (LR), Random Forest (RF), and Support Vector Machine (SVM) algorithms demonstrated notable precision in performance evaluations. The individual accuracy values for each algorithm are as follows: KNN, 86%; GBM, 86%; NB, 84%; LR, 89%; RF, 87%; and SVM, 87%. These results emphasize the importance of impedance features in the refinement of gesture recognition. The adaptability of the model was confirmed under different conditions, highlighting its broad applicability.

## Introduction

In the age of technological advancements, user interfaces have been continuously evolving to improve user experience and facilitate smooth interactions between humans and machines. Accurate interpretation and classification of finger gestures have emerged as a crucial aspect, particularly in the realm of human-computer interaction (HCI). Gesture recognition at the forefront of technological progress bridges the gap between machines and automatic communication. Although various techniques have been used to decipher this nonverbal language, the unwavering pursuit of precision, robustness, and userfriendliness remains. Optical and camera-based systems have historically dominated gesture recognition. Despite their widespread adoption, these modalities have limitations. They can be susceptible to environmental inconsistencies, such as ambient light, occlusions, and varied backgrounds, and the necessary external hardware, such as specialized cameras, can render these systems less integrated and cumbersome for users.

The study of intrinsic human signals for gesture recognition has attracted a great deal of attention recently. The human body, characterized by an intricate network of tissues, bones, and fluids, exhibits unique electrical properties that can be analyzed using impedance signals [1]. Unlike optical methods, ISSA explores internal bioelectrical signals that are relatively unaffected by external factors, offering a more consistent and natural approach to gesture detection. The impedance spectrum, which captures the resistance and reactance of tissues to alternating currents at different frequencies, undergoes variations as hand gestures alter the tissue configuration and composition. This suggests that each gesture may possess a distinct electrical “signature.” The widespread use of finger gestures as a means of communication, highlights the need for robust models to accurately decipher these gestures and ensure that machines can effectively and consistently interpret user inputs. The primary challenge lies in discerning between gestures that exhibit closely aligned morphological and resistive profiles because distinct finger gestures may share similarities in both physical appearance and electrical characteristics. This challenge is particularly pronounced in real-world scenarios, where subtle differences in these profiles must be accurately identified for precise gesture recognition.

The ability to effectively analyze impedance requires advanced computational techniques, owing to the complexity and noise present in such data. This underscores the potential of machine learning to improve the process of identifying gestures from impedance signals. Patterns and subtleties within these signals can be discerned using data-driven models, offering a robust gesture recognition mechanism [2]. Building on existing research in this area, this study explores the synergies between ISSA and machine learning, aiming to advance gesture-based interfaces and achieve greater accuracy and adaptability in gesture recognition.

Although previous studies have examined various aspects of gesture recognition, including hand gestures recognized using the input impedance variation of two antennas near the hand, EIT in the forearm, and surface electromyography (sEMG) signals from the forearm [3, 4, 5, 6, 7, 8, 9, 10, 11], a significant gap remains in the precise classification of finger gestures, particularly those with subtle but important differences in resistance under variable noise conditions. Consequently, this research aims to address this gap by evaluating the efficacy of several machine learning models in finger gesture classification and investigating the impact of signal-to-noise ratio (SNR) on classification performance. This study has two advantages. First, it assesses and contrasts the efficiency and robustness of multiple machine learning models in various noise situations. Second, it aims to uncover the intricate aspects and potential impediments present in resistive finger gestures, thus pinpointing prospects for enhancing the model performance and accurate gesture classification.

## Materials and methods

In the constant pursuit of advancing human-machine interaction, meticulous examination and detection of human gestures have emerged as a critical area of research, facilitating progress in fields such as robotics, healthcare, and assistive technologies. The core of this study involves the development of a novel methodology that seamlessly integrates the precision of impedance signals with the analytical capabilities of Machine Learning to create an enhanced system for accurate gesture recognition. By strategically placing electrodes on the arm, biometric signals based on variances in muscular and neural activity during gestures were carefully collected and subsequently subjected to rigorous analysis using a device that quantified the nuanced impedance associated with distinctive gestures. This impedance, which reflects the inherent electrical characteristics and variations in different gestures, served as the basis for our analysis.

**Figure 1:**
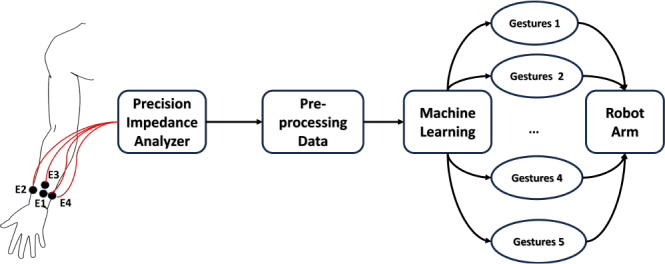
An overview of the gesture recognition system.

When impedance data are obtained, they are fed into advanced machine learning models that can identify complex patterns and nuances. These models can then classify and recognize various gestures with increased accuracy and effectiveness. The ultimate goal of this study was to apply these recognized gestures as intuitive, user-friendly, and nuanced controls to robotic arms, which can enhance their interaction and control granularity. This advancement represents a step toward achieving more natural, intuitive, and accessible human-machine interactive modalities, as illustrated in Figure 1. The following sections provide a detailed description of the methodology, experimental setup, data analysis, training, and evaluation of the machine learning model.

### Experimental setup

The primary objective of this research is to establish a comprehensive, precise, and designed experimental framework to exploit a robust data set by measuring the impedance signals derived from hand gestures. To achieve this, a strategic measurement protocol was devised that concentrates on crucial positions of the arm at distances ranging from 20 to 40 mm from the elbow, thereby ensuring the acquisition of inherently rich signals that reflect the anatomical and physiological intricacies involved in various gestures.

**Figure 2:**
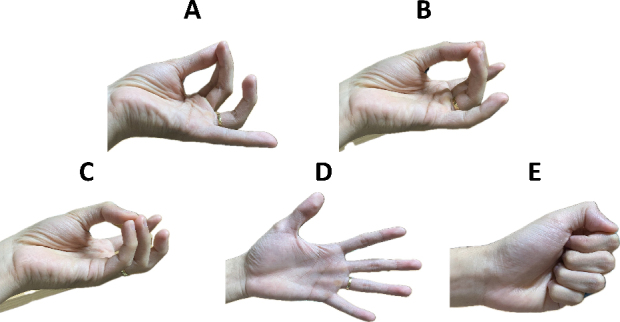
Evaluation of five hand gestures designed for holding, grasping, and relaxing activities.

The incorporation of the four electrodes served as a central aspect in the collection of signals, ensuring the accuracy and reliability of the obtained data. A comprehensive evaluation was conducted for five hand gestures, classified into three classes: finger-related movements and palm actions, such as relaxation and holding, as shown in Figure 2.

**Figure 3:**
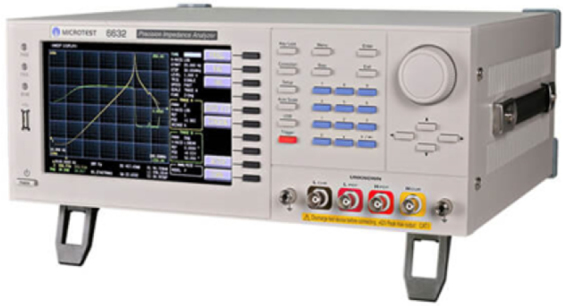
The precision impedance analyzer WK6632.

A critical component of the experimental design involved the use of the WK6632 precision impedance measurement device chosen for its precision and dependability to obtain impedance signals, as illustrated in Figure 3. The measurement frequency was set to 50 kHz, which allowed for an in-depth exploration of the impedance spectrum and facilitated a detailed analysis of the signals associated with each hand gesture. A constant input voltage of 100 mV was maintained throughout the measurement process to ensure the safety and comfort of human participants and the validity and reliability of the data obtained.

**Figure 4:**
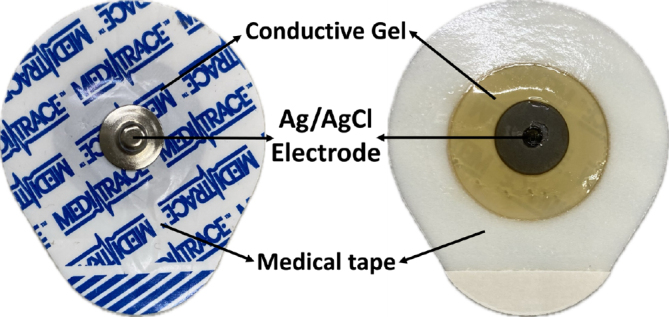
The electrode for gesture recognition data acquisition.

It is crucial to minimize the influence of contact impedance in real-world applications by maintaining a minimal contact impedance between the skin and electrodes. Additionally, consistent contact with the skin is necessary to reduce the variation in contact impedance when making gestures. Medically graded electrodes, known for their steady performance, were used to obtain stable and effective impedance data, which significantly improved wear comfort. This study did not investigate the impact of electrode size on impedance retrieval. The selected commercial medical electrodes consisted of an Ag/AgCl electrode encased in conductive gel, as illustrated in Figure 4. An adhesive material covers the gel, securing the electrodes and preventing them from falling during physical activity, thereby ensuring the stability of the measurement outcomes.

The current experimental setup served as the basis for subsequent data processing and analysis, guaranteeing that the machine learning models employed in the subsequent stages were trained, validated, and tested using a comprehensive and representative dataset. This approach improves the validity and applicability of the resulting gesture recognition system, thus ensuring that the models are effective and accurate. This approach is essential for the development of a successful system, and the dataset must be both diverse and representative of the gestures that are recognized.

### Data collection

To achieve precise and generalizable results in gesture recognition, it is essential to pay meticulous attention to data collection, which is a critical element that influences the subsequent analytical and modeling phases. In the initial phase of this study, the environment and conditions for data acquisition were controlled to mitigate potential interference or noise that could affect impedance measurements. This control ensured the fidelity and reliability of the signal in capturing the authentic physiological characteristics related to different gestures.

**Figure 5:**
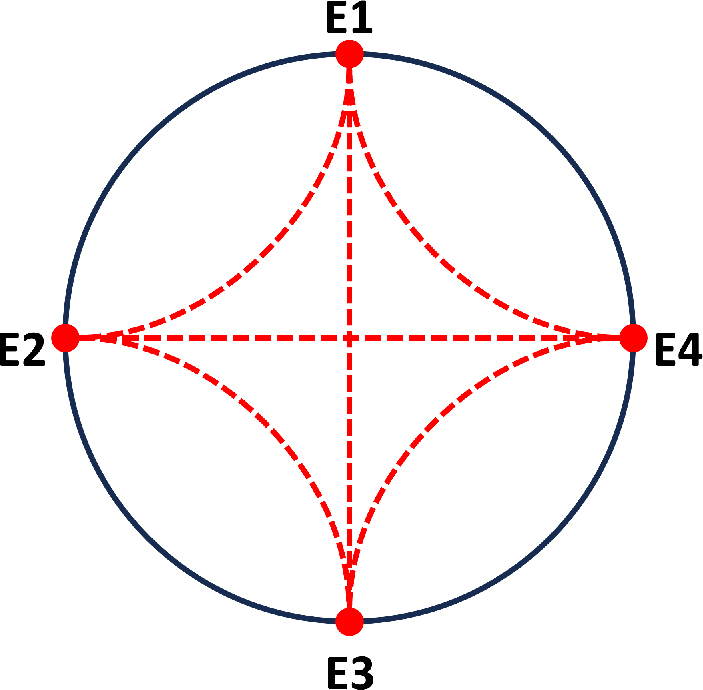
Electrode configuration for impedance data collection: using four electrodes (E1-E2, E1-E3, E1-E4, E2-E3, E2-E4, and E3-E4).

The signal acquisition strategy in this study involved the placement of four electrodes around the forearm as shown in Figure 5, including a cyclic process starting with electrodes 1 and 2. These two electrodes were fitted for injection of the current and signal measurements, respectively. This process then proceeds to electrode pairs 1-3, 2-3, 2-4, and 3-4. This arrangement, informed by preliminary studies that identified these locations as effective for discerning nuanced impedance variations among diverse gestures, encapsulated crucial variations within electrical impedance. These variations were consistent with the muscle and tendinous changes during gestures. The data are used to extract features of the interaction between different positions during changes in hand gestures and accentuate the variations in impedance signals through various gestures and electrode placements. A WK6632 precision impedance measurement device was used, and the experiment adopted a frequency of *50kHz*.

All participants were fully informed of the study and provided their written informed consent before participation. Participants were instructed to perform a set of five basic gestures that represented fundamental activities. The experiments were conducted 200 times for each gesture and were divided into four sessions, with each session spaced five minutes apart. This deliberate spacing allows the muscles involved in performing the gestures to rest fully between sessions, reducing the risk of fatigue and ensuring more consistent and reliable measurements. By spacing out the repetitions in this manner, any potential muscle fatigue or performance degradation that could affect the measurement accuracy was minimized. This procedure facilitated the identification of systematic patterns and anomalies, which were instrumental in subsequent analyses and model training.

The collected data were judiciously allocated to facilitate rigorous training, validation, and testing of the machine learning models. Based on widely accepted ratios, the data were apportioned into training, validation, and testing sets to mitigate overfitting and objectively evaluate the performance of the model on unseen data. Data partitioning was performed as follows:

- Training set: 70% of the total data was allocated for modeling training.
- Validation set: 10% of the data was reserved for fine-tuning the model parameters and preventing overfitting.
- Testing set: The remaining 20% of the data was set aside to objectively evaluate the performance of the model and its generalizability to unseen data.

The training and validation datasets were from four out of five volunteers, and the testing dataset was from the remaining volunteers. Data segmentation offers a systematic structure for model development and assessment, ensuring that the results and performance indicators derived from the testing set accurately reflect the potential usefulness and effectiveness of the model in practical situations.

A metric known as the signal-to-noise ratio (SNR) [12] is of paramount importance in determining the reliability or reproducibility of the measurements. It measures the consistency of the results obtained from repeated measurements under identical conditions. Previously, SNR was determined by calculating the ratio of the average signal value to the noise level across the measurement channels, as expressed in Equation 1:

1 $${\text{SNR}}_{i}=\frac{{\bar{v}}_{i}}{SD{\left[v\right]}_{i}}$$

where *SD*[*v*]_*i*_ represents the amplitude of the noise derived from the standard deviation of numerous measurements for each respective channel, and ${\bar{v}}_{i}$, represents the signal amplitude estimated from the mean value of these multiple measurements per frequency. This calculation provided a crucial estimate of the reliability and consistency of the gathered data.

### Pre-processing data

In this study, Principal Component Analysis (PCA) was promptly applied to effectively navigate the inherent dimensionality and complexity of the data, determining the fundamental characteristics that underlie gesturespecific variances [13]. The mathematical formulations outline the core mechanics of PCA in the context of its implementation. Assuming a data matrix X of size (m × n), where m is the number of observations (measured impedance at each frequency) and n is the number of variables (distinct frequencies), the first step involves centering the data by subtracting the mean impedance value for each frequency, mathematically represented as Equation 2:

2 $$X_{\text{centered}}=X-\bar{X}$$

where $\bar{X}$ denotes the mean of *X* across all the observations.

The covariance matrix *C* of the centered data was calculated to understand how each pair of variables covaried, providing insight into the inherent patterns in the dataset represented by Equation 3:

3 $$C=\frac{1}{m-1}X_{\text{centered}}^{⊤}X_{\text{centered}}$$

The subsequent computation of the eigenvalues (λ) and eigenvectors (*V*) of the covariance matrix provides the foundation for the formation of the principal components.

4 CV=λV

In Equation 4, each column vector *v_i_* in *V* signifies a direction in the original data space and its associated eigenvalue λ_*i*_ denotes the variance of the data when projected onto *v_i_*.

The principal components are derived through the linear transformation of the centered data *X*_centered_ with eigenvectors *V*, leading to a new data matrix *Y* as shown in Equation 5:

5 $$Y=X_{\text{centered}}V$$

To ensure optimal retention of features with minimal dimensions, component selection is typically governed by the cumulative explained variance (CEV) criterion in Equation 6:

6 $$\text{CEV}=\frac{\sum_{i=1}^{k}{\text{λ}}_{i}}{\sum_{i=1}^{n}{\text{λ}}_{i}}$$

where *k* denotes the number of PCs chosen. Typically, *k* is selected such that CEV ≥ 0.95, which ensures that at least 95% of the total variance is retained.

The clear distinction between gestures along PC1, as opposed to PC2 and PC3, can be attributed to the significant variance in impedance data captured by PC1. This variance closely aligns with the most distinct features observed among the gestures. Essentially, PC1 embodies the principal variance within the dataset, highlighting the key differences in forearm impedance triggered by various hand gestures. This indicates that PC1 predominantly encapsulates the significant disparities between gestures, thereby serving as the crucial axis for gesture classification.

When examining the roles of PC2 and PC3 in the classification process, it becomes evident that although their contributions enhance overall classification accuracy, their influence is notably lesser than that of PC1. This diminished impact stems from PC2 and PC3’s role in capturing data variations which, despite their relevance, do not exhibit as clear a distinction among the gestures under analysis. Thus, while they offer additional insights that refine the classification process, they are not the primary drivers of differentiation.

In the exploration of classification models, the assessment included the impact of incorporating PC1, PC2, and PC3 as input features, evaluating them both as a collective and on an individual basis, within the model optimization framework. The selection of principal components was approached as a hyperparameter during the training phase, facilitating the determination of the most effective combination for enhancing classification accuracy. The findings reveal that including PC2 and PC3, notwithstanding their relatively minor separation, plays a critical role in achieving optimal accuracy for the recognition of different hand gestures through forearm impedance analysis.

In this study, PCA-enhanced impedance data, encapsulated within selected principal components, were forwarded to machine learning models, providing a computationally efficient but potent substrate that retained crucial variance and patterns vital for robust and accurate gesture classification. This enhances the predictive potency of the model and alleviates computational intensity and the tendency to overfit.

### Model selection and training

This study of gesture recognition focused on the use of machine learning models to navigate the complexities and nonlinearities of the bioelectrical impedance data. It uses an ensemble of models, including Naive Bayes, Gradient Boosting Machine, Support Vector Machine, Random Forest, k-nearest Neighbors, and Logistic Regression [14, 15, 16, 17, 18, 19]. Each model was selected for its ability to handle the multidimensional and varied nature of the data, ensuring a comprehensive and robust evaluation and comparison.

Each model in the study was chosen to harness its unique attributes and capacities in learning patterns and to classify the multidimensional data derived from electrical impedance:

- Naive Bayes (NB): Esteemed for its simplicity, NB was employed due to its efficacy in highdimensional spaces and its inherent probability-based classification mechanism, aligning seamlessly with the data’s characteristics.
- Gradient Boosting Machine (GBM): GBM was adopted for its prowess in optimizing misclassifications and its robustness against overfitting, even amidst complex data landscapes.
- Support Vector Machine (SVM): Recognized for its capacity to discover non-linear boundaries, SVM was utilized to manage the intricacies of the highdimensional feature space.
- Random Forest (RF): RF, with its ensemble learning method, was utilized to foster both model robustness and accuracy, mitigating risks associated with overfitting.
- k-Nearest Neighbors (KNN): KNN was implemented to exploit its non-parametric nature and capability to discern nuanced differences amidst the closely knitted gesture classes.
- Logistic Regression (LG): LG, while traditionally linear, was engaged for its interpretability and ability to provide probability estimates, underpinning a benchmark against which the other models could be evaluated.

### Metrics

The dataset was divided into three sets: training, validation, and testing sets. This process was designed to maintain the integrity and impartiality of a model’s development and evaluation. Implementing stratified k-fold cross-validation ensures that the unique characteristics of each gesture class are equally represented and evaluated throughout the training and validation phases as outlined in Equation 7 [13].

7 $$\begin{array}{ccc} D_{\text{train}}=\left(1-p\right)D, & \text{and} & D_{\text{valid/test}}=pD \end{array}$$

where *D* signifies the dataset and *p* is the percentage of data allocated for validation and testing.

Upon starting the training process, the models were extensively evaluated against an unseen testing set to enhance their performance. This evaluation was facilitated by employing a variety of metrics, including accuracy, precision, recall, and F1-score, which were calculated using Equations 8, 9, 10, and 11. These metrics provide a comprehensive assessment of a model’s competence, enabling the selection of the most efficient model to classify gestures with both precision and recall accurately.

8 $$\text{Accuracy}=\frac{TP+TN}{TP+TN+FP+FN}$$

9 $$\text{Precision}=\frac{TP}{TP+FP}$$

10 $$\text{Recall}=\frac{TP}{TP+FN}$$

11 $$F1\text{-Score}=2\times \frac{\text{Precision}\times \text{Recall}}{\text{Precision}+\text{Recall}}$$

### Informed Consent

Informed consent was obtained from all subjects involved in the study.

### Ethical approval

The research related to human use has been complied with all relevant national regulations, institutional policies and in accordance with the tenets of the Helsinki Declaration, and has been approved by the authors’ institutional review board or equivalent committee.

## Results

**Figure 6:**
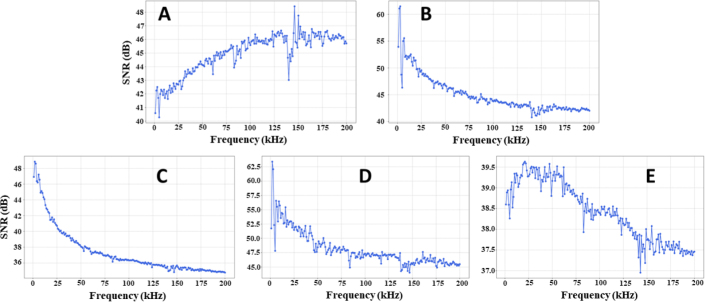
Comparative SNR performance graph for five hand gestures (A, B, C, D, and E), with each gesture measured for 208 values (04 electrodes) and conducted with 200 repetitions.

In examining the SNR within the acquired impedance data corresponding to different gestures, observations were made that potentially impact model development and application efficacy. SNR, a crucial metric that indicates signal quality and discernibility in potential noise, was calculated for each gesture among various participants, electrode placements, and conditions, as shown in Figure 6.

In general, all gestures exhibited an SNR greater than 40 dB, which is a benchmark often considered indicative of high-quality signals amid potential electrical and environmental noise. This elevated SNR underscores the efficacy and reliability of the experimental setup and data acquisition methodology, reinforcing the validity and reproducibility of subsequent analyses and findings from this dataset. An SNR greater than 40 dB is typically deemed sufficient for many biological signal processing applications to ensure the discernibility and analyzability of genuine physiological signals amid potential noise.

Despite the overall high SNR, variations in the SNR were observed between gestures, providing nuanced insights into the inherent signal characteristics and potential challenges of different gestures. The “C” gesture displayed the lowest SNR in the repertoire, still surpassing the 40 dB threshold, ensuring its viability for data analysis and application. However, the comparatively lower SNR requires a cautious and rigorous approach in the subsequent data processing and modeling phases, ensuring that the model adequately captures and generalizes the subtle signal variations and patterns intrinsic to this gesture without succumbing to potential overfitting or misclassification.

The relative decrement in SNR for the “C” gesture may be attributed to its inherently subtle and less distinct muscle and tendon activity compared to more pronounced gestures, yielding a less prominent impedance variation and signal amidst inherent noise. This scenario requires skilled handling in model development, potentially involving more intricate and adaptive algorithms or specialized preprocessing techniques to effectively navigate and harness the subtler nuances of this gesture.

**Figure 7:**
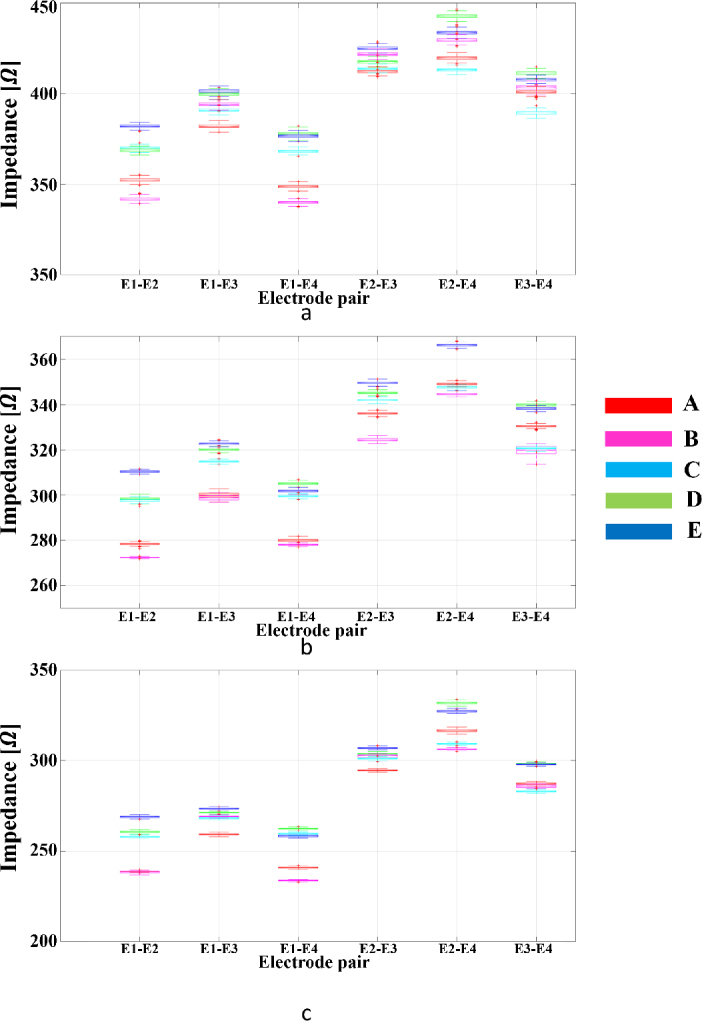
The measurements obtained from electrode pairs across various gestures for three volunteers with 5 gestures A, B, C, D, and E: (a) Person 1, (b) Person 2, (c) Person 3.

Notable uniformity was observed in most gestures regarding the magnitude of the impedance (|*Z*|) within the investigated frequency spectrum. The respective |*Z*| graphs in varying gestures converged to analogous attitudinal values at the corresponding frequencies, demonstrating a broad constancy in the bioelectric resistance encountered during different hand movements. This consistency permeated across the entire explored frequency range of 50 kHz, with the impedance magnitudes delineating a congruent trajectory for the studied gestures. The standard deviation value for the dataset was calculated and is shown in Figure 7. Figure 10 illustrates the broad constancy in electrical resistance encountered during different hand movements.

**Figure 8:**
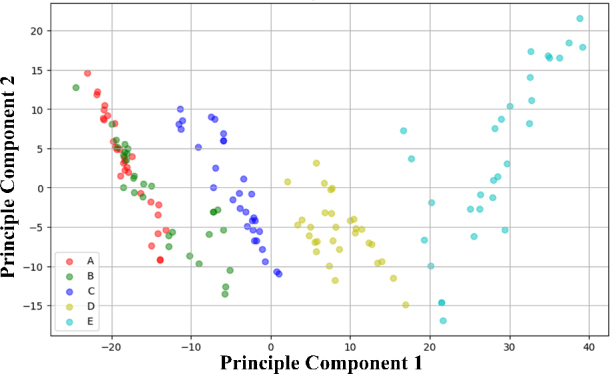
Investigating PCA for gesture recognition with 2 dimensions.

**Figure 9:**
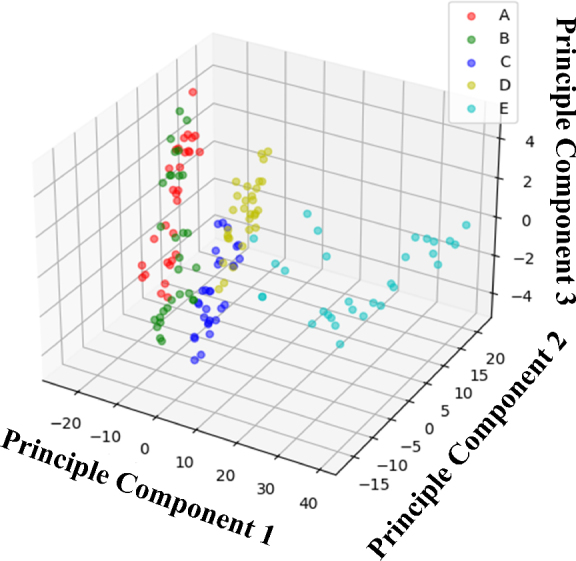
Investigating PCA for gesture recognition with three dimensions.

**Figure 10:**
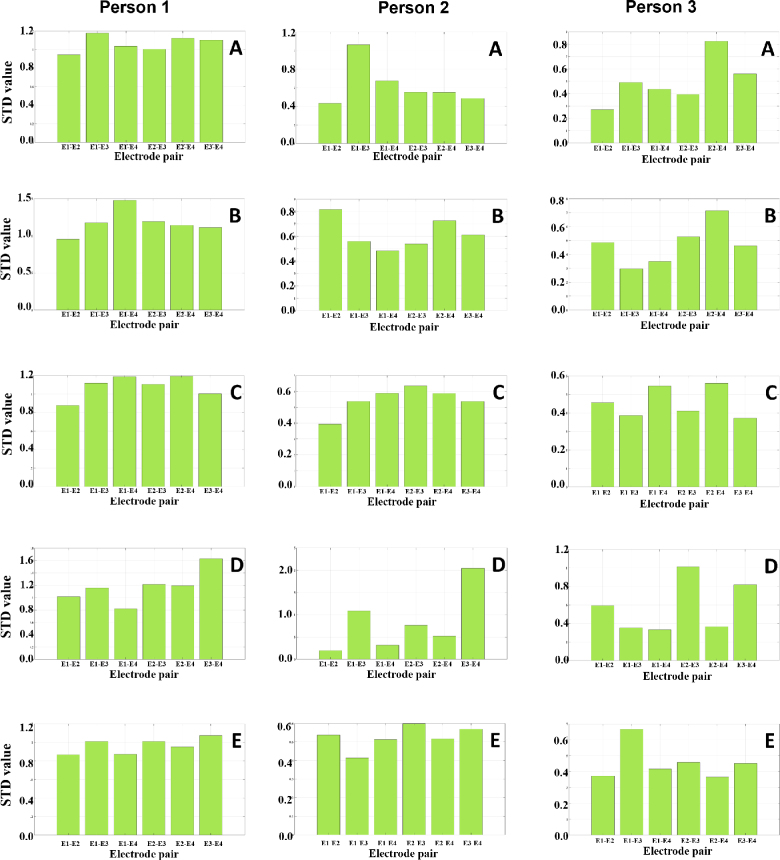
Standard deviation values for sample data from 3 volunteers with labeled gestures A, B, C, D, and E for Person 1, Person 2, and Person 3.

Analyzing the two-dimensional and three-dimensional PCA spaces, as shown in Figures 8 and 9, respectively, and derived from the impedance (*Z*) values, provided insight into the unique characteristics that distinguish the evaluated gestures. These visualizations, generated using the first three principal components (PC1, PC2, and PC3), which collectively captured a substantial portion of the variance in R values across various gestures, revealed a discernible clustering pattern within the PCA space, confirming the effectiveness of R values in distinguishing between different gestures. Although some gestures formed tight clusters, indicating a degree of uniformity in resistive profiles among subjects (e.g., gestures A, B, and C), others exhibited more dispersed and expansive distributions (e.g., gestures D and E), suggesting inherent variability in how individuals express resistive characteristics for specific gestures.

The presented results delineate the comparative performance of six distinct classification algorithms–KNN, GBM, Naïve Bayes, LR, Random Forest, and SVM–evaluated using a 5-fold cross-validation strategy. In Table 1, a preliminary overview of the mean precision reveals that the Logistic Regression model achieved the highest average accuracy of 89%, while the other models produced slightly lower averages: notably, Random Forest and SVM (87%), KNN (86%), and GBM (86%). Nonetheless, a nuance resides in evaluating the model’s robustness and reliability, which can be gauged by observing consistency across individual folds.

**Table 1:** Performance evaluation of machine learning models.

Model	Fold	Accuracy	Precision	Recall	F1-Score
KNN	Fold-1	0.70	0.72	0.70	0.72
	Fold-2	0.93	0.96	0.93	0.92
	Fold-3	0.91	0.92	0.91	0.90
	Fold-4	0.94	0.96	0.94	0.94
	Fold-5	0.81	0.87	0.81	0.79
	Mean	0.86	0.89	0.86	0.85
GBM	Fold-1	0.94	0.96	0.94	0.94
	Fold-2	0.93	0.94	0.93	0.92
	Fold-3	0.76	0.63	0.76	0.68
	Fold-4	0.95	0.95	0.95	0.95
	Fold-5	0.70	0.79	0.70	0.69
	Mean	0.86	0.86	0.86	0.84
Naive Bayes	Fold-1	0.72	0.73	0.72	0.71
	Fold-2	0.93	0.96	0.93	0.92
	Fold-3	0.96	0.97	0.96	0.96
	Fold-4	0.91	0.95	0.91	0.89
	Fold-5	0.70	0.74	0.70	0.67
	Mean	0.84	0.87	0.84	0.83
LR	Fold-1	0.80	0.79	0.80	0.78
	Fold-2	0.94	0.96	0.94	0.94
	Fold-3	0.93	0.94	0.93	0.92
	Fold-4	0.93	0.95	0.93	0.92
	Fold-5	0.85	0.89	0.85	0.83
	Mean	0.89	0.90	0.89	0.88
Random Forest	Fold-1	0.74	0.74	0.74	0.73
	Fold-2	0.93	0.96	0.93	0.92
	Fold-3	0.96	0.97	0.96	0.96
	Fold-4	0.93	0.96	0.93	0.92
	Fold-5	0.81	0.89	0.81	0.79
	Mean	0.87	0.90	0.87	0.86
SVM	Fold-1	0.72	0.73	0.72	0.71
	Fold-2	0.94	0.96	0.94	0.94
	Fold-3	0.94	0.95	0.94	0.94
	Fold-4	0.94	0.96	0.94	0.94
	Fold-5	0.80	0.86	0.80	0.78
	Mean	0.87	0.89	0.87	0.86

Despite its simplicity, the KNN algorithm exhibits a mean precision of 86%, although it reveals variability between folds ranging from 70% to 94%. This fluctuation suggests sensitivity to the choice of the neighbor parameter or potential overfitting, necessitating an in-depth exploration of the hyperparameters and distance metrics.

The GBM model, while achieving exemplary performance in certain folds (94% and 95% in folds 1 and 4, respectively), showed susceptibility to fluctuations (76% and 70% in folds 3 and 5, respectively). This variability may arise from inadequate handling of outliers, noise, or suboptimal tuning of hyperparameters, such as the learning rate and tree depth, which warrants further investigation for future refinement.

NB and SVM demonstrated proficient mean performance (84% and 87%, respectively), showing robustness across folds. The consistent accuracy of the SVM of more than 72% across all folds underscores its effectiveness in managing the feature space. However, fine-tuning the kernel parameters can further enhance performance.

The LR model earned a reputation as a top performer, consistently delivering high-accuracy results across all folds. Its reliable precision, recall, and F1 scores attest to its dependability and robustness regardless of the data distribution and the set of features.

RF, known for its resistance to overfitting and its ability to manage complex data structures, exhibits impressive performance, with consistently high scores (74% to 96%) across multiple folds. Its potent precision and recall metrics demonstrate balanced errors and proficiency in managing biases and variances. Within the decision landscapes of these models, a notable pattern of misclassification emerges, particularly between the finger gestures “A” and “B.” The subtle morphological differences and closely spaced resistance of these gestures pose a challenging problem for classifiers, as evidenced by the disproportionately high misclassification rates within these categories across all models.

**Figure 11:**
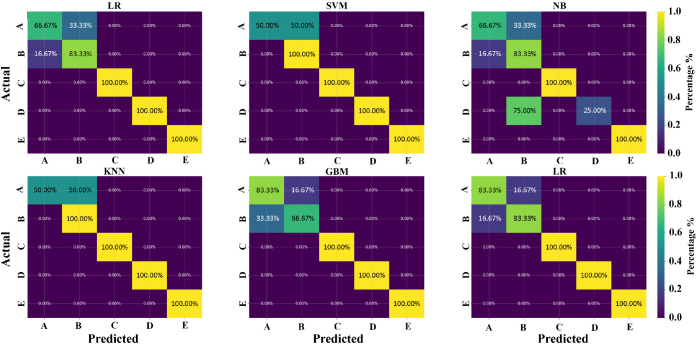
Confusion matrices demonstrating the classification accuracy of machine learning models for hand gesture recognition.

Examining the confusion matrices (Figure 11) reveals a recurring pattern, where the gesture “A” is frequently misclassified as “B” and vice versa. This difficulty is substantiated by the varying misclassification rates among gestures in the different models. In the KNN model, 50% of “A” gestures were misclassified as “B.” In the GBM model, a significant misclassification occurs with 16.67% of the “A” gestures misidentified as “B” and 33.33% of “B” gestures misclassified as “A.” Naïve Bayes displays a distinct pattern, with 33.33% of “A” gestures misclassified as “B” and 16.67% of “B” gestures misclassified as “A,” as well as 75% of “D” gestures misclassified as “B.” The RF, LR, and SVM models exhibited different misclassification patterns, highlighting the intricate challenge of accurate classification in various model architectures.

While the intrinsic similarities in the resistive gestures “A” and “B” contribute to this confusion, a deeper analysis of their loading scores in PCA space further elucidates the dilemma. PCA, effectively reducing the dimensionality of the data set, reveals the clustered proximity of gestures “A” and “B”, especially in reduced two- and three-dimensional spaces, amplifying the challenge of unambiguously distinguishing between them using linear models.

To provide further support, the principal components, which are primarily influenced by lower-spectrum frequencies, show a notable separation in the resistive characteristics of most gestures. However, they face challenges when confronted with the nuanced disparities between gestures “A” and “B”. Morphological similarity, evident in closely aligned resistive profiles, leads to a conflation of identities, as reflected in the PCA loadings, demonstrating overlap or proximity in the PCA space. This trend suggests the need to explore more complex nonlinear models or integrate additional features, potentially influenced by temporal aspects, to better distinguish between these two gestures.

## Discussion

Impedance measurements in the forearm during hand gestures are influenced by various physiological factors, including geometrical changes in the muscles and tendons, variations in electrode distance, and contributions from the skin. These factors are critical for the accurate interpretation of our findings. Muscular activities during gestures lead to changes in the cross-sectional area through which electrical currents pass, thereby affecting the impedance measurements. Additionally, electrode positioning and pressure on the skin can vary slightly with muscle movement, potentially affecting the electrode distance. Skin properties such as sweating and tension also contribute to the measured impedance. While these factors are peripheral to muscle and tendon changes, they collectively influence the impedance readings during different gestures.

Examining the signal-to-noise ratio (SNR) in the acquired impedance data for various gestures has yielded valuable insights that affect the development and effiicacy of the model. The SNR, which represents the signal quality in the presence of potential noise, was calculated for the participants, electrode placements, and conditions. While all gestures surpassed the 40 dB SNR benchmark, indicating high-quality signals, variations across gestures, particularly for “C” and “E,” imply challenges in subsequent modeling.

As the SNR decreases, the models struggle with obscured gesture signals, compromising the accuracy and clustering within the PCA space. Lower SNR scenarios, such as those for “C” and “D,” require careful processing and potentially more adaptive algorithms. The uniformity of the impedance magnitude between the gestures highlights the shared electrical pathways. Challenges in low-SNR scenarios highlight opportunities for model improvement using denoising techniques or additional features.

The implementation of PCA in both 2 and 3 dimensions provides insightful perspectives on the nuances of gesture classification within the dataset. Upon analyzing the loading scores, it becomes apparent that lower spectrum frequencies predominantly influence PC1, which is crucial to separate gestures with marked differences at lower frequencies. On the contrary, PC2 and PC3 were more significantly influenced by mid- and high-frequency R values, indicating their role in dissecting finer resistive disparities among gestures.

The detection of anomalies within the PCA space revealed outliers that typically arise from gestures perceived as biomechanically awkward or challenging. This observation is reflected in the increased variability within these specific gesture clusters, which provides a discernible pattern of performance inconsistency related to the biomechanical demands of the particular gestures.

The multivariate nature of PCA not only facilitates dimensionality reduction but also illuminates characteristics pertinent to discriminating between diverse gestures. This streamlined approach improves subsequent classification efforts and interpretative clarity regarding the influential attributes of the principal extracted components. A comprehensive evaluation of gesture data considering both primary and subtle features underscores the utility of PCA for capturing and emphasizing data variability and gesture uniqueness.

While PCA provides insights, its linearity may limit complex, non-linearly separable gesture clusters, as observed in gestures “A” and “B”. Future investigations could explore advanced dimensionality reduction techniques such as t-SNE or UMAP. These findings emphasize the complexity of finger gesture classification, particularly for closely aligned gestures.

The classification results from KNN, GBM, Naive Bayes, and SVM offer insights into these challenges and prospects. Variations in the accuracy of gestures underscore the complexity of automating nuanced gesture classification. Different models exhibit varied capabilities, highlighting the challenges of consistent recognition, especially for closely related gestures.

The use of non-gel electrodes in wearable applications offers numerous advantages such as enhanced comfort, durability, and user-friendliness. Unlike gel electrodes, which require preparation and maintenance, non-gel electrodes allow for hassle-free attachment and easy cleaning, promoting long-term usability and adoption. In addition, they may help alleviate skin irritation or allergic reactions, thereby enhancing user acceptance and compliance. Opting for non-gel electrodes creates a practical and accessible gesture recognition solution suitable for everyday use.

In comparison with Electrical Impedance Tomography (EIT), this method focuses on utilizing forearm impedance data acquired with a smaller electrode setup, providing simplicity and ease of use. While EIT offers superior spatial resolution, it typically requires a more extensive electrode array and complex instrumentation. However, EIT may offer richer spatial information, enabling a more detailed analysis of muscle activation patterns during gestures. Regarding surface electromyography (sEMG), the impedance-based approach captures broader physiological changes associated with gestures, including alterations in muscle geometry and tissue conductivity. Although sEMG provides detailed information on muscle activity, it may necessitate precise electrode placement and signal processing techniques to differentiate between individual muscle contributions. In contrast, this method offers a more comprehensive view of forearm physiology, potentially complementing sEMG in gesture recognition.

This developmental model highlights the need for further research and refinement. Future work may explore alternative models, hybrid approaches, or advanced feature extraction. Incorporating various gestures and user input can enhance recognition capabilities. Practical adaptations and iterative advances based on model insights aim to increase the efficacy of finger-gesture classification models in real-world applications.

## Conclusions

This study aimed to investigate the intricate domain of finger gesture classification using machine learning models and explore the dimensionality and structure of gesture data using PCA. Various models, including K-Nearest Neighbors, Gradient Boosting Machine, Naive Bayes, Support Vector Machines, Random Forest, and Logistic Regression, have been employed to determine the nuanced differences in the resistive profiles of gestures. This approach provides valuable information and highlights the challenges inherent in classification tasks.

PCA effectively illuminates the capabilities and limitations of linear models in distinguishing between gestures. It offers a perspective on the variability and structure within the data, while underscoring the difficulties in discerning closely related gestures. Examining signal quality through SNR and morphological similarities between gestures has emerged as a significant factor that influences classification accuracy, particularly in real-world scenarios characterized by prevalent noise.

The results of this research lay the foundation for subsequent studies, suggesting potential pathways and methods, such as diving into non-linear dimensionality reduction techniques, implementing advanced noise mitigation strategies, or incorporating deep learning approaches. Practical applications of accurate and reliable finger gesture classification span various domains, from assistive technologies to interactive gaming, making this research a foundational step toward developing technology that is adept at intuitively understanding human gestures. Future studies can further build on these findings, refine and expand current knowledge and approaches to finger gesture classification, and enhance the interface between technology and human communication.
